# Functional Imaging of Chemically Active Surfaces with Optical Reporter Microbeads

**DOI:** 10.1371/journal.pone.0136970

**Published:** 2015-09-02

**Authors:** Punkaj Ahuja, Sumitha Nair, Sreenath Narayan, Miklós Gratzl

**Affiliations:** Department of Biomedical Engineering, Case Western Reserve University, Cleveland, Ohio, United States of America; Queen's University at Kingston, CANADA

## Abstract

We have developed a novel approach to allow for continuous imaging of concentration fields that evolve at surfaces due to release, uptake, and mass transport of molecules, without significant interference of the concentration fields by the chemical imaging itself. The technique utilizes optical “reporter” microbeads immobilized in a thin layer of transparent and inert hydrogel on top of the surface. The hydrogel has minimal density and therefore diffusion in and across it is like in water. Imaging the immobilized microbeads over time provides quantitative concentration measurements at each location where an optical reporter resides. Using image analysis in post-processing these spatially discrete measurements can be transformed into contiguous maps of the dynamic concentration field across the entire surface. If the microbeads are small enough relative to the dimensions of the region of interest and sparsely applied then chemical imaging will not noticeably affect the evolution of concentration fields. In this work colorimetric optode microbeads a few micrometers in diameter were used to image surface concentration distributions on the millimeter scale.

## Introduction

Current approaches to imaging concentration fields at surfaces include different modalities of scanning probe microscopy where a microscopic probe is raster-scanned along the surface of interest. Scanning electrochemical microscopy, SECM, introduced by Engstrom and initially explored by Bard utilizes an ultramicroelectrode as the scanning probe to acquire electrochemical concentration measurements at precise positions over a substrate within the region of interest, ROI [1,2]. Data points of a completed scan are compiled to generate a discrete concentration map within the entire ROI which then can be processed to obtain a continuous map. SECM has been utilized for a wide variety of applications including chemical analysis of biological processes at single cells and cell monolyers as well as at 3D constructs [3–7]. Concentration mapping has also been performed with fluorescence confocal laser scanning microscopy where fluorescent dyes are dissolved in the bulk solution and used to visualize pH or H_2_O_2_ profiles at surfaces [8,9]. Scanning probe techniques have, however, some limitations. Most importantly the time needed to complete a scan can be longer than what the dynamics of the studied process would ideally require, and the moving tip can potentially induce microflow especially at faster scan rates [10]. Laser scanning is typically used to assess very small ROIs.

Kopelman and others have designed nanometer scale Photonic Explorers for Biomedical use with Biologically Localized Embedding, or PEBBLEs, for chemical measurements in single cells and multicellular constructs [11–16]. PEBBLEs, delivered into cells by non-specific endocytosis, address several of the limitations associated with conventional ‘free’ intracellular fluorescent dyes [12]. PEBBLEs, however, have not been used in extracellular contexts such as in studies on cellular secretions because of technical limitations: many particles would float away from the cells’ surface while others would non-specifically endocytose.

We describe here Optode-Bead based Chemical Imaging, OBCI: an approach to functional imaging of chemically active surfaces that has features in common with scanning probe microscopy as well as PEBBLEs. Optically active microbeads are used for reporting concentrations at specific locations on top of the studied surface. The microbeads are a few microns in size and therefore cannot be endocytosed if cell surfaces are studied. Further, the beads are entrapped and immobilized in a very thin hydrogel layer and thus they cannot float away. Concentrations are measured at each bead and a contiguous concentration profile is then computed by post-processing as in SECM. However, OBCI enables the acquisition of a chemical image of the entire ROI instantaneously while eliminating the possibility for microflow that could distort the concentration field.

Further features that distinguish OBCI from SECM and the PEBBLE approach include the size of the reporter beads. Compared to PEBBLEs which are nanoscale particles, the optode microbeads are larger, with diameters on the micron scale which implies that larger features of mass transport, on the order of 50 μm and beyond, can be captured. However, the accessible ROI can also be larger, typically up to 5 mm. The distribution of beads is random, and therefore post-processing is more involved than in SECM. However, to increase the number of concentration data points and thus improve spatial resolution of the chemical image only requires more (and possibly smaller) beads to be immobilized in the hydrogel membrane which does not impact the temporal resolution of imaging. Moreover, OBCI can be used for imaging concentration fields *above* a surface, or *below* a construct (imaging from underneath the preparation). The resulting map provides information on the movement and local accumulation and depletion of molecules of interest via pre-calibration of the beads.

Optode beads can encode concentration to fluorescence intensity or to color. In this work colorimetric microbeads are used. Calibration of color is more robust than that of intensity, because color is determined by a spectrum, whose shape does not depend on the intensity of illumination, or on the thickness of the optically active region, in this case the dimensions of the optode beads. In practical reflectance imaging typically 3 color values (red, R; green, G; blue, B) represent the spectrum at each bead and their intensities relative to each other define color that is related to concentration.

Various types of chemically sensitive optical beads are available using optode chemistries and can be made with sensitivity to different ionic species and other analytes including sodium[17], potassium [17], ammonia [18], sulfur dioxide [19], hydrogen sulfide [20], ethanol [21], acetone [21], oxygen [22,23], carbon dioxide [24], and metabolites such as glucose [25]. This enables OBCI to be applied to study a variety of processes.

In this work we visualize pH propagation from a pore. A theoretical description of pH propagation is problematic with common diffusion equations. Contrary to diffusion of other ionic species, pH propagation is a remarkably complex process due to a variety of factors. First, hydrogen and hydroxide ions are dissociation products of water: the medium itself. Compared to the movement of other ions and molecules in this medium, pH propagation consists not only of diffusion on the molecular scale, but also titration at every location, restricting the use of typical mass diffusion equations. Further complicating the situation are spatial variations of the original pH and buffer capacity that may exist. This is why we use the term pH (or acid/base) propagation rather than diffusion in this work. If there are multiple sources, sinks, and preexisting variations in other concetrations or ionic strength then a theoretical approach is hardly feasible. For example, variations in pH at a live epithelial cell monolayer due to ionic secretions is not known because of a lack of a suitable pH-mapping technique.

This article describes OBCI and its application to the visualization of pH maps in a dynamic system where a microscopic pore acts as a source of acid or base. The pore can be thought of as mimicking a biological pore or other small source. Utilizing the optode-bead based chemical imaging approach with pH sensitive beads enables pH propagation of the acid or base coming from the pore to be imaged. OBCI can provide a unique solution that can be extended to applications where current techniques are limited due to extraneous scanning time or non-specific uptake of the measurement probes.

## Materials and Methods

### Materials

To make the cocktail for the preparation of the optode microbeads 9-(diethylamino)-5-[(2-octyldecyl)imino]benzo[a]-phenoxazine (hydrogen ion selective chromoionophore III), Bis[(12-crown-4)methyl]-2-dodecyl-2-methylmalonate (bis(12- crown-4)), sodium tetrakis[3,5-bis(1,1,1,3,3,3-hexafluoro-2-methoxy-2- propyl)phenyl]borate (NaHFPB), bis(2-ethylhexyl)sebacate (BEHS), poly(vinyl chloride) (PVC) were purchased from Sigma-Aldrich (St. Louis, Missouri, USA). Type 1 agarose was used for all membranes (Sigma-Aldrich). Agar membranes were prepared from 0.0001 M phosphate buffered saline, PBS, with pH 7.4 at 25°C containing 0.138M NaCl and 0.0027M KCl. Deionized water (specific resistance >18.2 MΩ cm) by a Milli-Q water system (Millipore Corp., Bedford, MA, USA) was used in all solutions.

### Apparatus

**Fig 1:**
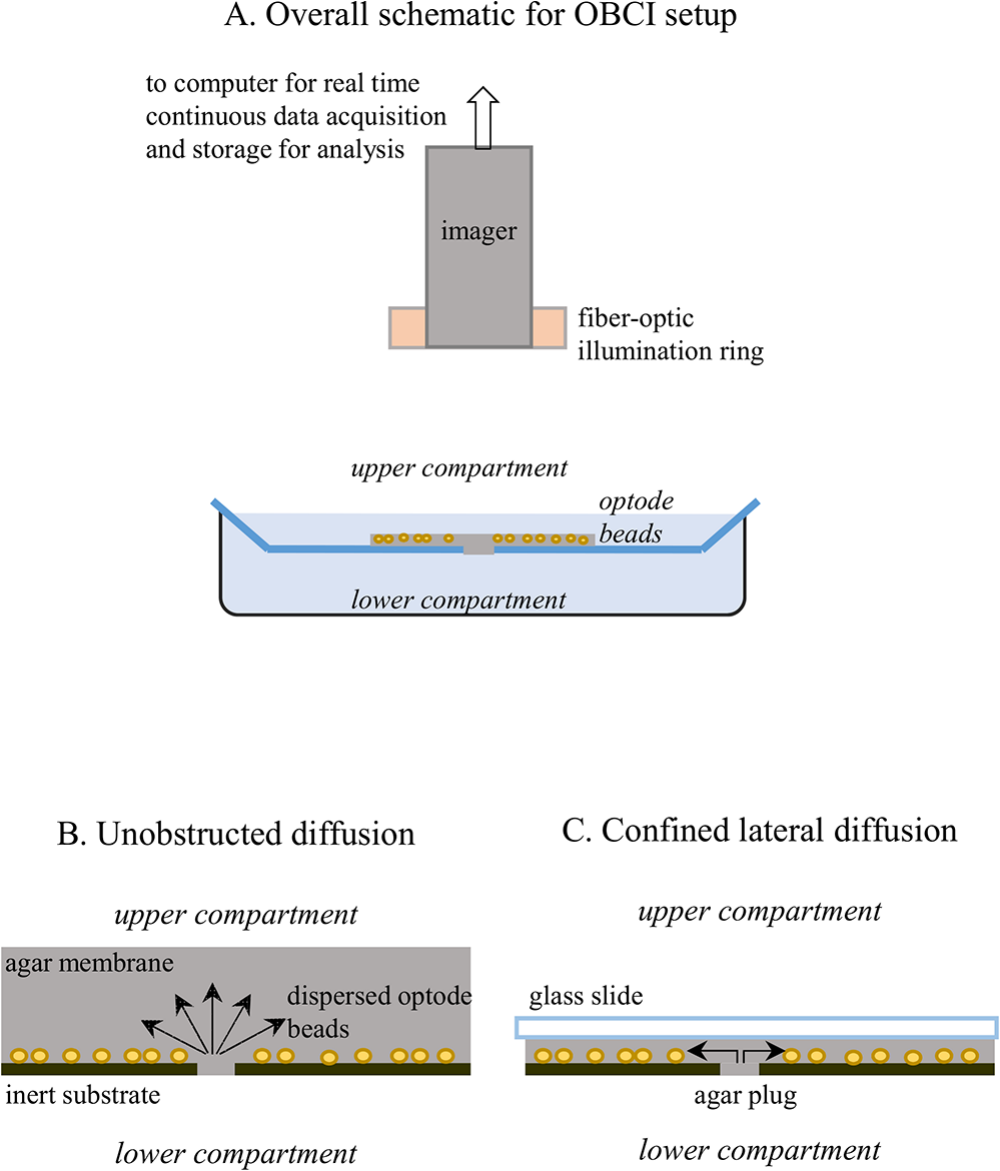
Experimental setup for microbead-based optochemical imaging of pH diffusion above an inert substrate.

The overall experimental setup is shown schematically in Fig 1, panel A. Reflectance color images were acquired from above the dish where pH propagation was occurring using a color digital microscope camera (CFW-1012C, Scion Corporation, USA) and ImageJ software (NIH, Bethesda, Maryland, USA). A circular microscope light was attached to the camera and used as a ring shaped illumination source throughout experiments (Imagelite Model 20, Stokeryale, USA). This arrangement provided uniform illumination over the entire 2mm2 ROI. Mass transport is shown schematically for 3D hemi-spherical diffusion in Panel B and 2D cylindrical diffusion in panel C. Acid/base diffusion is dynamically imaged with optode microbeads as shown in Fig 1 in each panel. A polystyrene weigh dish (41 x 32 x 8 mm, Fisher Scientific, Waltham, Massachusetts, USA) was used as an inert substrate in diffusion experiments. Agar membranes containing immobilized, dispersed optode beads were placed on the upper surface of the weigh dish. The dish was used also as the upper reservoir. A small micropore was made across the weight dish and filled with agar to form a plug. This pore was used as the microscopic source of the acid or base reagent diffusing from the lower compartment into the upper compartment where imaging took place. The weigh dish was fixed on top of a plastic petri dish (Falcon, 60 x 15 mm style, Becton Dickinson, Franklin Lakes, New Jersey, USA), which acted as the lower reservoir containing the buffer whose pH would propagate through the pore.

Images were acquired from above the upper reservoir using a color digital microscope camera (CFW-1012C, Scion Corporation, USA), using ImageJ software (NIH, Bethesda, Maryland, USA). A microscope light was attached to the camera and used as the illumination source throughout experiments (Imagelite Model 20, Stokeryale, USA).

### Preparation of microscopic optode beads dispersed in agar membrane and calibration

pH sensitive optode beads 1–3 μm in diameter were made using the spray-dry method described previously by Tohda et. al [25]. Small loose aggregates were also spontaneously formed from some individual beads. The prepared optode particles were manually dispersed on a glass slide using a pulled glass capillary tip. A second glass slide was pressed to the first slide, to disrupt the aggregates and for further dispersion of the beads by static charges. The slides were separated and two pieces of aluminum foil, 7 μm thick, were placed at the edges of the slides to act as spacers. The two slides were then clamped together using small binder clips placed over the pieces of foil. One of the open ends of the clamped structure was placed in warm, aqueous 1 wt% agar and the liquid gel layer was wicked between the slides with pH sensitive optode beads dispersed throughout by capillary action. (It is noted that for some applications such as in cellular studies aggregates may need to be more perfectly dispersed than what was necessary in this work.)

The 7 μm membrane with dispersed optode beads was soaked in PBS for 30 min. The beads were then exposed to PBS solutions with adjusted pH values between 5.0 to 8.0 in 0.5 pH increments. Solutions were made by adjusting 0.1M PBS with HCl (0.01 M) and KOH (0.01 M) stock solutions, respectively. A pH glass electrode was used to ensure accurate pH values (Accumet 925 pH meter, Fisher Scientific, USA). The calibration was performed by first going in the basic direction of the pH scale from PBS (pH ~7.36) to 7.5 and 8.0, and then in the acidic direction in 0.5 pH units to pH 5.0. The calibration was then repeated from acidic to alkaline direction. The membrane was exposed to the respective solution for 30 minutes and images were taken approximately 1 minute intervals determine color response and response time. Images were acquired using the Scion microscope camera described above.

### Preparation of the substrate with a pore filled with an agar plug

A pore in the polystyrene weigh dish was formed by placing a hot soldering iron near a needle tip (Talon American, Stamford, Connecticut, USA) and placing the hot needle tip perpendicular to the polystyrene substrate. A small pore through the substrate (in the range of 150 to 210 μm in diameter in this work) was formed. 1 wt% agar was made in 0.01 M PBS diluted four times, adjusted to pH 3.5 or 8.5. The pH of the solution was adjusted with HCl or KOH. The substrate was placed upside down approximately 10 cm above simmering DI water. A drop of agar was placed on the substrate covering the source and was allowed to sit above the hot water vapor for 10 min to fill the plug with agar.

### Preparation of agar membranes for immobilizing dispersed optode beads

For lateral diffusion experiments a 7 μm agar membrane with dispersed beads was prepared as described above and then a slide was placed on top. For hemi-spherical diffusion experiments the slide was not used. A thicker, 100 μm agar membrane using a 100 μm thick spacer (microscope cover slip, Fisher Scientific) was prepared, to immobilize the beads at the substrate. The beads were dispersed as described above. The agar membranes do not limit the rate of acid/base mass transport because small ions diffuse across the pores of agar essentially like in free buffer [26].

### Experimental setup for mass transport and OBCI measurements in acidic and basic direction

To visualize the propagation of acidic pH 1x10^-4^ M PBS was adjusted to pH 3.5 for the lower reservoir and pH 7.5 for the upper reservoir. The optode-bead-containing agar membrane was placed flush with the substrate over the agar plug in the upper reservoir. A glass slide was used above the membrane during lateral diffusion experiments with a 1 g weight was placed on the top corner of the glass slide covering the membrane, outside of the ROI, to hold it in place. For diffusion of basic solution a 1x10^-4^ M PBS solution was adjusted to pH 8.5 for the lower reservoir and pH 4.5 for the upper reservoir. The membranes were treated as described for acid diffusion.

### Image analysis for calibration

ImageJ was used to measure red, green, and blue, RGB, intensities at 10 different regions where beads were found in each image. To account for ambient lighting variations in image acquisition, each RGB value was normalized using Pythagorean normalization as described above (Eq 1).

### Image analysis for visualization of the mass transport process

The scion microscope camera and ImageJ software were used to acquire images of the dispersed beads immobilized on top of the substrate. Images were acquired in 30 second intervals throughout the length of each experiment.

For each acquired image, the end goal is to generate a concentration map using the information of the color of the beads. This requires a multi-step process which includes 1) extraction of data by thresholding, 2) Delaunay triangulation, 3) color averaging using circular symmetry, and 4) pixel normalization. These are described below.

Matlab was used for image processing. To obtain data with better temporal continuity, linear interpolation in time was performed on consecutive images to obtain data for 10 second intervals rather than the acquired 30 second intervals.

In order to extract color data from the beads from each image a thresholding scheme was developed to obtain a mask of the image for beads. This would allow analysis using the beads’ color information, and utilize this information to fill in the pixels where no beads were placed. The mask included beads (data) and excluded the white substrate. The resultant was a mask of the image consisting of logical variables, where pixels which represented beads were displayed as white = 1, and background/substrate displayed as black = 0. This map was used for Delaunay triangulation [27] for each image in the same experiment.

To produce a color at each pixel of the image, the color information of each bead was required from the unprocessed images. Using the mask generated by thresholding, each image was processed in Matlab [28] using the GridData function. A Delaunay triangulation was done as an interpolation method to fill in the points where no data was collected and to resolve data from discrete points and an. The Delaunay triangulation maximizes the minimum angle of all angles of the triangles to avoid skinny triangles. The GridData function provided a Delaunay triangulation for each image using only bead data corresponding to the mask. Each image was first decomposed to three stacks, a Red, Green and Blue stack, respectively. The triangulation was calculated for each of these stacks. The stacks were then compiled once again to produce a color image.

The Delaunay triangulation provides information at most pixels based on data from beads. However, the result shows sharp changes at the vertices of the triangles made by the interpolation function. In order to smooth the image, circular symmetry around the source was assumed around a circular source. Color-averaged rings were then calculated growing outward from the source. Each ring included an average color of its own diameter, 5 pixels wide and also the adjacent outer 5-pixel ring and inner 5-pixel ring, thus creating a 5 pixel ring with 15-pixel radially averaged information. These rings began at the source and were extended throughout the entire image.

## Results and Discussion

### Optode-bead based chemical imaging of pH propagation at an inert substrate from a micropore

The propagation of acid or base from a source cannot be described as simple diffusion of H^+^ or OH^-^ ions: diffusion and titration occur simultaneously at every position in the concentration field, which also implies that both ions diffuse in the same time towards each other at both sides of the boundary between acidic and basic pH. Additionally, the ion product of water must hold at every point in space at all times. This tightly links the movement of H+ and OH- ions everywhere. Buffer capacity also has its own field and local titration depends on this, plus the actual pH at the given point in space. Therefore the loci (typically, a surface) of neutral pH propagate in a more complicated way than what could be expected for simple ion diffusion, since it depends on both the actual pH and buffer capacity fields. This makes it difficult to predict the evolution of pH fields without actually visualizing them in experiments. A somewhat analogous problem would be diffusion of an ion in a solution where its chelator is also present.

pH propagation from a single pore (in the range of 150–210 μm in different experiments) at an inert substrate was visualized under different conditions. Diffusion into an open solution space from the pore above the substrate (Fig 1A) results in a 3D hemi-spherical diffusion pattern. By mounting a transparent inert cover above the substrate at a distance that is much shorter than the dimensions of the region of interest, ROI (Fig 1B), 2D lateral diffusion with circular symmetry can be visualized. Similar transport patterns cannot be studied with microelectrode scanning since the narrow diffusion layer (7 μm in this work) cannot be accessed by an electrode. The evolution of pH fields above a 2-mm^2^ area is visualized here. ROIs of similar size are often used in scanning probe techniques, in studies on cell clusters and other applications [29].

To visualize propagation of acid or base pH-sensitive optode microbeads (1–3 μm in size) are dispersed on top of the substrate. A sparse dispersion is made (average distance between nearest beads: 50–100 μm) in order to minimize obstructions to the propagating concentration fields. Many beads were distributed also in aggregates which were small enough relative to the size of the pore source and the dimensions of the ROI, to provide with a pH map of sufficient resolution. Further, in this paper though aggregates existed, we use only the term “optode microbeads” since they are the probes that actually respond to pH including in the aggregates.

Interpolation of color between the beads and radial symmetry of the diffusion process around the pore in both the 3D and the 2D diffusion schemes were used for image processing to obtain color at each pixel of a snapshot where there are no beads. Pixel colors can be transformed into a pH field at each time point using pre- or post-calibration of the beads.

### Color response analysis

The red, green and blue, RGB, reflectance intensity values obtained at pixels where optode beads were located in the images can be used in various ways to quantitatively represent the color information that is linked to pH where a bead is located.

**Fig 2:**
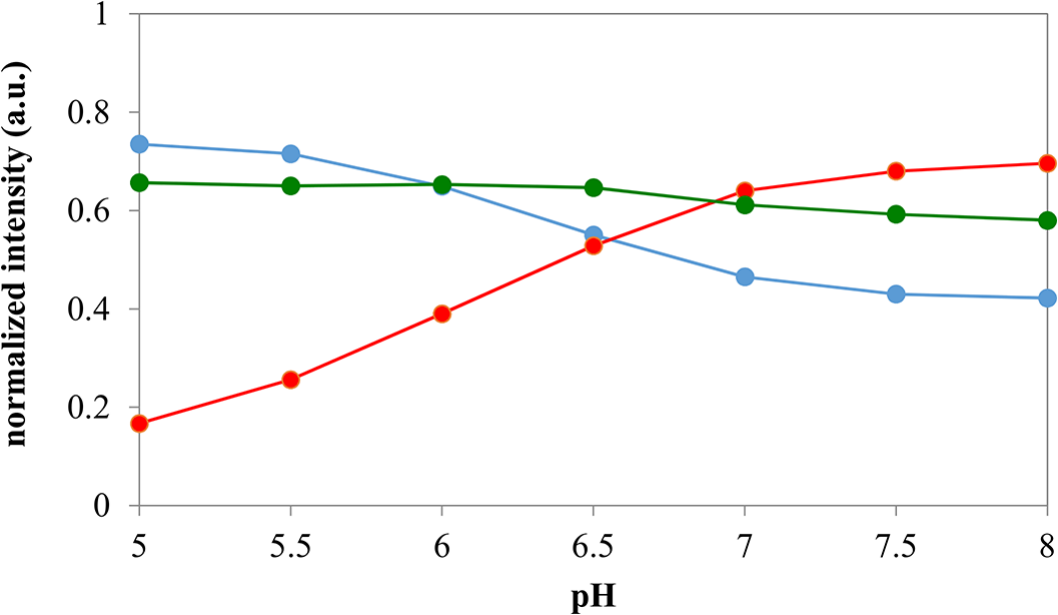
Calibration of pH-sensitive optode beads.

The beads respond to pH with gradual color change from blue to orange as the pH changes from 5 to 8, sufficient to map pH propagation in this work. A calibration is shown in Fig 2 after Pythagorean normalization of the data. The sensitivity of red color intensity, R, is the greatest. Blue intensity is about half as sensitive as red. The response in green color to pH is relatively flat, which could allow it to be used as internal reference. The greatest rate of change is around pH 6.5, below neutral pH with a resolution of about 0.07 pH unit. Therefore the grayish green color at pH 6.5 is used in this work to track pH propagation, though the loci of pH 6.5 are slightly displaced relative to the loci of neutral pH.

Theoretically, either of the three color components could be used for pH calibration. However, it is advantageous to normalize the RGB values before data analysis. This is to avoid interference from eventual variations in the intensity of the light source or sensitivity of the detector. To avoid also potential interference from spectral variations in the illumination and/or detector, the RGB values could be further normalized to a color standard area such as a white reference [25]. This, however, was not necessary in these experiments due to the stable light source and camera used.

The common method of RGB normalization is normalization to the sum of the RGB values [30]. This transforms the RGB values such that they always lie between 0 and 1. However the absolute value (length) of the corresponding 3D vector [R, G, B] will also vary between 0 and 1 depending on the actual color seen.

In order to be able to use vector analysis for color the measured intensity of each color component was divided by the square root of the sum of squares:

$$X_{Pn}=\frac{X}{\sqrt{R^{2}+G^{2}+B^{2}}}$$ (1)

where *X* is *R*, *G*, or *B* and *Pn* stands for Pythagorean normalization. As opposed to the most common method of normalization to the sum of the color components, Pythagorean normalization always adjusts the RGB vector to unit absolute value, and thus the actual composite color can be perceived as the direction of a vector of unit length in a 3D coordinate system. The color vector defined this way will always end at a point on the positive eighth of the surface of a unit sphere in the normalized RGB coordinate system. Since the response of an optode system is color, the intensity information is not needed, which is why a vector of unit length is sufficient for calibration and measurement. (We note that the “hue” variable is also often used to quantify color [31], which is especially useful when there is significant background. However in this work there was very little background in the RGB values.)

The color information that is obtained with a regular RGB camera is encoded in 3 values. After normalization only 2 independent variables remain. Thus, for example R_Pn_, or B_Pn_, or both could be used for data analysis. The ratio of these values is also useful, which simplifies the use of calibration since only one value’s dependence on pH needs to be considered that could be potentially more sensitive than either value alone. However this ratio value will not be limited to the 0–1 range: it will not be a “bound” variable.

### Dynamic color distributions of dispersed optode beads during acid and base propagation

**Fig 3:**
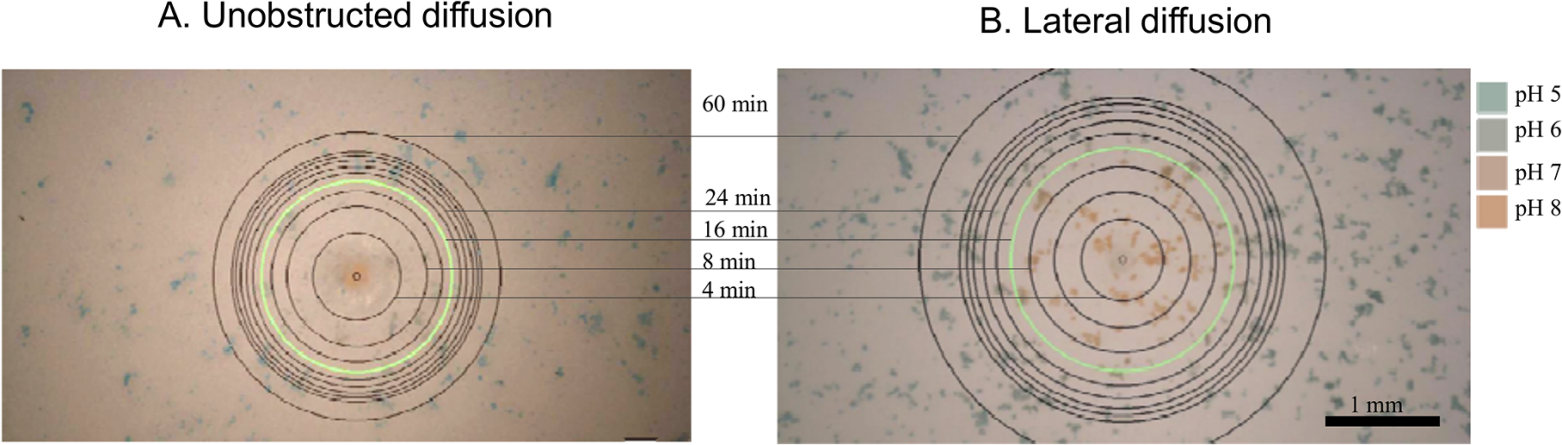
Propagation of base from a pore 205 μm in diameter (n = 10) imaged at the substrate in free space (Panel A) and in a laterally confined space (Panel B).

In the experiments shown in Fig 3 the pH was 8.5 in the lower compartment and the pH was 4.5 in the upper compartment. Both solutions were 10^−4^ M PBS whose pH was adjusted to the respective values. Hemi-spherical (left panel) and lateral (right panel) propagation of base at multiple time points are shown in Fig 3. The images illustrate the spreading of alkaline pH from the pore at the surface. Similar images have been obtained using an acidic source compartment, visualizing the propagation of acid pH (not shown). The spreading of orange color from the pore represents the outward movement of alkaline pH, while the retracting blue color represents progressive over-titration of the originally present acidic solution. The intermediate color between blue and orange is grayish green. The green circle in both panels shows a front of about pH 6.5 (from the calibration in Fig 2) at the time the image shown was acquired. The black circles indicate the spreading of this pH 6.5 front over time. Therefore they do not exactly coincide with the circle of neutral pH (not shown) but they indicate the dynamics of the process correctly.

In the experiments where the spreading of acid pH into an alkaline compartment was visualized similar trends were observed (not shown). Compared to the results shown in Fig 3, acid propagation was faster than the propagation of base. At 25°C, the respective diffusion coefficients are D_H+_ = 9.31 x 10^−5^ cm^2^/s [26], and D_OH-_ = 5.27 x 10^−5^ cm^2^/s [32]. Further, the pH of the strongly acidic reagent used was 3.5, and that of the target compartment, 8.5, was mildly basic. These circumstances rationalize the observation that acid propagation was seen in both cases to be faster than base propagation.

### Evolution of continuous color maps over time

**Fig 4:**
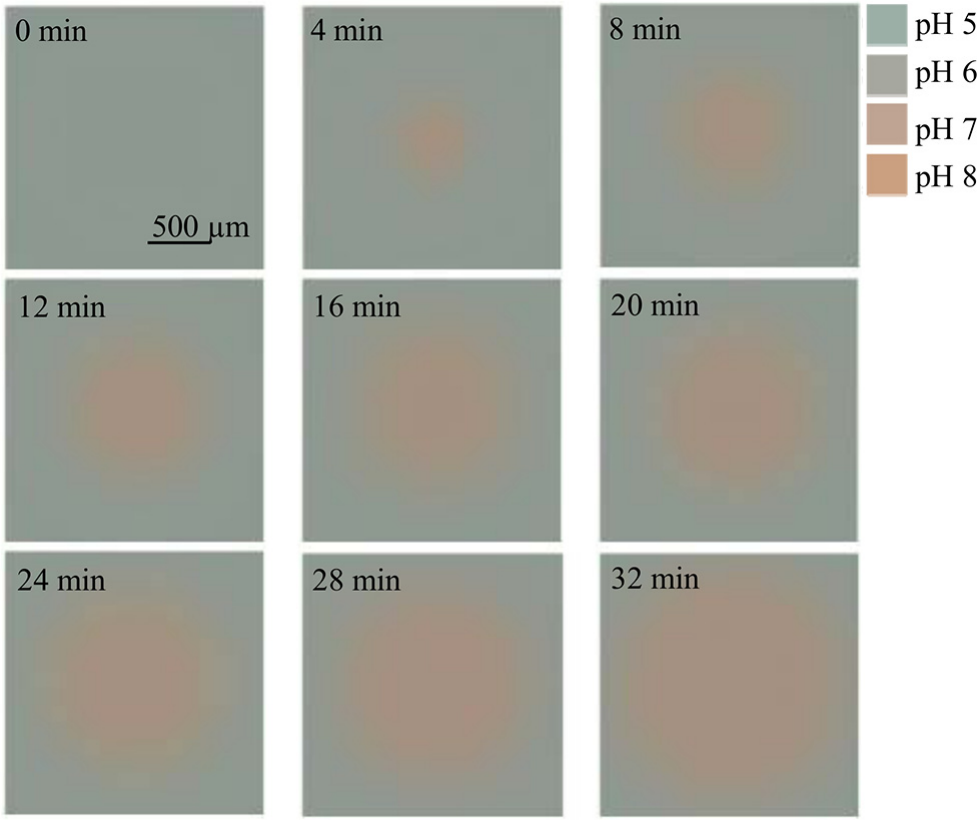
Continuous color maps of lateral base propagation from a pore 165 μm (n = 10) in diameter at the substrate.

**Fig 5:**
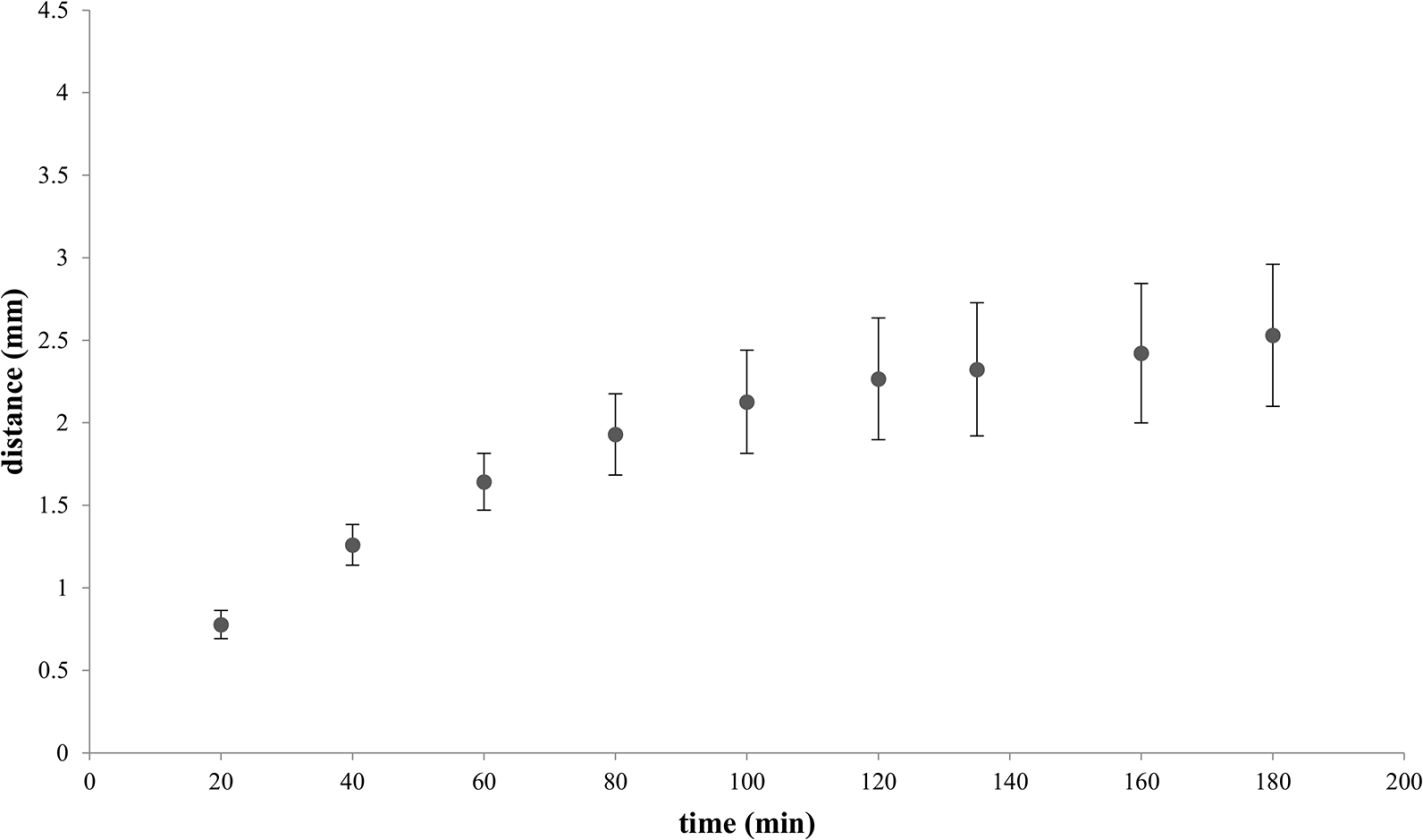
Distance of the pH 6.5 circle from a pore 165 μm in diameter (n = 10) over time in lateral pH propagation at the substrate. The results represent the averages of 6 consecutive measurements.

Fig 4 shows continuous color maps developing over time in a lateral diffusion experiment where hydroxyl ions are entering the upper compartment from the pore and spread in 2D. The continuous maps have been obtained after image processing (see Experimental). In obtaining continuous maps, we take into account two frequencies of interest, temporal and spatial. The successive images represent color coded pH fields. In Fig 5 the evolution of the distance of the locus of pH 6.5, corresponding to respective circles in Fig 3 is plotted versus time.

In the experiment shown in Fig 3A the propagation of base, after gradually slowing down, stopped at a distance of about 1.25 mm from the pore (not shown). This steady state condition is reached in about 90 min. The following considerations illustrate that pH propagation is a very different process than simple molecular diffusion in water.

At a distance a few times larger than the radius of the pore the diffusion patterns become essentially hemi-spherical around the source. At the final steady state of 3D radial diffusion a hyperbolic concentration profile is expected:

$$c(r)\approx c_{0}(R/r)$$ (2)

where R is the radius of the pore, r is distance from the center of the pore (r > R), and c_0_ is concentration in the pore [33]. The concentration of OH^-^ in the pH 8.5 buffer decreases 100 fold when reaching the pH 6.5 line. Thus, at steady state transport the pH 6.5 line should be at 100 times the radius of the pore: r_st_ = 100 R ≈ 10 mm. However the expansion of the base|acid front in the experiment whose early part is shown in Fig 3A, stops at 1.25 mm. Further, one can observe that the entire pH profiles for both unobstructed and lateral propagation are essentially flat over most of the distance from the pore, instead of the sharp hyperbolic decay that diffusion theory predicts. This is because pH propagation is the simultaneous movement and dissociation-association of the two constituent ions of water: the medium itself as opposed to the movement of a single molecule driven alone by its concentration gradient.

Information contained in Figs 3–5 could not therefore be obtained from theoretical considerations for reasons described further above, and supported by our experimental findings. In brief, the data obtained in this work are the result of interactions between complex processes that have never been mathematically interpreted and therefore an equation that would reproduce the pH maps and in the same time have physical meaning, is not available. This and other insights can, however, be obtained with optode bead based chemical imaging, the technique introduced in this work.

### Immobilization of optode beads and their pH response

To ensure that the beads are distributed relatively evenly and that they remain in the same place throughout the experiment, they were immobilized at the bottom of a thin membrane made of agar gel. Since diffusion rates of small ions in water are similar to diffusion in low-concentration agar gel, the membrane does not obstruct the process was found in our work and experimentally by others [26,34]. A membrane of ~7 μm placed between the substrate and a transparent, inert cover slip was used in the 2D diffusion experiments, and a ~100 μm membrane placed on top of the substrate was used in the experiments involving 3D diffusion. The beads were immobilized at the bottom of this gel layer at the studied surface.

Beads a few microns in diameter respond to a sudden pH change in the order of seconds. Small aggregates of beads also respond fast enough (less than 10 s) to pH change to be able to consider the response practically instantaneous relative to the rate of diffusive spreading over the entire ROI used in this work, during experiments in the order of 60 minutes.

### Dynamic color distribution during pH propagation in 2D and 3D

The rate of spreading decreases over time in both the hemi-spherical and the lateral experiments. However, this decrease is more pronounced in hemi-spherical pH propagation than in the lateral process. Also, the circles propagate significantly farther from the pore in lateral diffusion compared to the hemi-spherical case during the same time period. These observations can be rationalized by considering that in the left panel a 3D space is titrated while in the right panel the target compartment is essentially 2D: a cylindrical enclosure with microscopic height. Thus, in the first case the volume increment to be titrated during a given time increment is proportional to r^2^ while in the lateral setup, to r where r is the radius of the circles measured from the center of the source pore. Thus, the amount of reagent required for titrating a volume increment at the same radius is much larger in the hemi-spherical case than in the lateral arrangement.

### OBCI compared to scanning chemical imaging techniques

Different modalities of scanning probe microscopy, SPM, have allowed chemical imaging of numerous types of active surfaces and have had applications to a variety of processes of scientific interest [1,8,10,35]. There are some general limitations with the scanning approach, however. The time needed to accomplish a dynamic experiment with multiple scans may be prohibitive. Another problem may be associated with the rate of change to be monitored that may be faster than the temporal resolution that one complete scan allows. Also, the need for simultaneous control of distance of the probe from the substrate may require two independent measurement schemes to be integrated, such as scanning of a microelectrode probe and electrochemical processing, and another measurement scheme to assess the distance of the probe from the substrate for distance control. This is the case in many SECM experiments, except when the same microelectrode probe is used for both the electrochemical measurement and assessing distance using electrochemical impedance spectroscopy [36]. Lateral hydrodynamic drag of small volumes of the solution by the scanned microprobe can cause flow artifacts in SECM. This can induce local mixing that modifies the gradients of the concentration field, and may also add an apparent isotropic component to any anisotropic concentration field.

Microbead-based OBCI is a noninvasive technique for imaging chemical activity at surfaces that provides snapshots of the entire ROI therefore avoiding any of the issues mentioned above. Virtually continuous image acquisition is possible over the entire ROI, thus producing simultaneous 2D snapshots. Scanning in SPM can take long enough time for the actual concentration field to change significantly during one complete scan. This will introduce distortions in the reconstructed image. In OBCI, the only limitation in measurement speed is that the response of the beads must be faster than the process imaged. Further, continuous, smooth images can be reconstructed at each time point after image post-processing. The resulting concentration profiles represent the entire ROI as they evolve in time. The optochemical microbeads (optode or other type) also provide continuous, reversible responses to changes in concentration in the order of seconds or less. This allows for virtually continuous visualization in time over the entire ROI. The setup for OBCI is miniature, compared to scanning techniques which require an x-y-z positioning and scanner system of high precision, a potentiostat or millivoltmeter, and often additional equipment for distance control. OBCI can also be used in additional applications where a microprobe cannot be scanned due to physical constraints, such as within a microscopic gap like in the lateral transport experiments in this work, or under 2D or 3D cell constructs placed on a substrate or filter [37]. The dispersion of optochemical beads allows for microprobes to be placed directly onto the imaged surface for measurements without the need for distance control.

Optode beads are custom made. However they are fabricated in a relatively simple fashion using a spray dry method which, after dispersing eventual aggregates, provides consistent, uniform size of beads and high yield [25]. Further, by making the beads custom, the user is able to adjust the ratio of sensing molecules to enable sensitivity in given concentration ranges of interest. Finally, recently developed methods using a solvent displacement approach provide alternative, straightforward methods for bead fabrication [38]. This can enable additional variations in bead characteristics such as size or material.

In many experimental settings, however, only a scanning probe approach is feasible, for example because of the lack of a suitable optical reporter bead. Also, a large variety of analytes and distance scales can be covered with existing scanning techniques. Scanning speed and associated temporal resolution, as well as spatial resolution in many settings are sufficient with scanning probe microscopy. This includes visualization of processes at single biological cells with sub-micrometer resolution [29] which is hardly possible with beads a few micrometers in size. Optochemical imaging using microbead dispersions is therefore a complementary technique to scanning probe microscopy.

## Conclusions

Microbead-based optochemical imaging, OBCI, entails the use of a sparse dispersion of optically active microbeads that act as reporters for imaging chemical fields as they evolve in time at different surfaces. Continuous image acquisition combined with post-processing image analysis allows for complex chemical transport processes to be mapped continuously in the presence of sources and sinks across an entire surface and over time. It is possible to minimize interference of the beads with mass transport of the analyte by maintaining sufficient free surface areas between beads, and/or using very small beads. By functionalizing beads for specific molecules of interest, this method can be used for a variety of applications.

Required bead size depends on the size of the smallest features to be discerned in the imaged chemical field. Beads a few micrometers in size are large enough for color imaging. The color of submicron sized beads become faint and beads below the diffraction limit of the light used can no longer be imaged with clarity.

The technique described here is not about measuring different analytes for analysis but rather, to monitor their movement and propagation, reactions, generation and uptake at surfaces using colorimetric imaging. Problems that can be addressed with this approach include non-biological processes such as corrosion, or diffusion at surfaces that contain sources and sinks, as well as inhomogeneous and/or reactive concentration fields. Biological applications include secretion processes at the apical side of oriented cell monolayers, and above, inside, or under 3D cell constructs.
